# Patient and site characteristics associated with pirfenidone and nintedanib use in the United States; an analysis of idiopathic pulmonary fibrosis patients enrolled in the Pulmonary Fibrosis Foundation Patient Registry

**DOI:** 10.1186/s12931-020-1315-4

**Published:** 2020-02-10

**Authors:** Colin H. Holtze, Elizabeth A. Freiheit, Susan L. Limb, John L. Stauffer, Karina Raimundo, Wayne T. Pan, Kevin R. Flaherty, Hyun J. Kim

**Affiliations:** 1 Department of Internal Medicine, Division of Pulmonary & Critical Care Medicine, University of Michigan, North Campus Research Center, 2800 Plymouth Rd, SPC 2800, Ann Arbor, MI 48109 USA; 2 Department of Biostatistics, University of Michigan, Ann Arbor, MI USA; 3 Genentech, Inc., US Medical Affairs, South San Francisco, CA USA; 4 BioMarin, San Rafael, CA USA; 5 Department of Medicine, Division of Pulmonary, Allergy, Critical Care and Sleep Medicine, University of Minnesota, Minneapolis, MN USA

**Keywords:** Idiopathic pulmonary fibrosis, Therapeutics, Clinical decision-making

## Abstract

**Background:**

Pragmatic use of the anti-fibrotic medications pirfenidone and nintedanib for idiopathic pulmonary fibrosis (IPF) in the United States (US) has not been studied and may be different from international settings due to structural differences between health care systems. This study examined the relationship between patient- and site-level characteristics and anti-fibrotic (a) use and (b) selection.

**Methods:**

Data from the Pulmonary Fibrosis Foundation Patient Registry was used to perform univariable and multivariable regressions with generalized linear mixed models. A random effects model examined registry site variation.

**Results:**

703 of 1218 (57.7%) patients were taking a single anti-fibrotic of which 312 (44.4%) were taking nintedanib and 391 (55.6%) were taking pirfenidone. Up to 25% of patients using an anti-fibrotic may have been excluded from clinical trial participation due to having too severe disease as measured by diffusion limitation for carbon monoxide. Age (OR = 0.974, *p* = 0.0086) and diffusion capacity of the lungs for carbon monoxide (per 10% increase in percent-predicted; OR = 0.896, *p* = 0.0007) was negatively associated with anti-fibrotic use while time (in log of days) since diagnosis (OR = 1.138, *p* < 0.0001), recent patient clinical trial participation (OR = 1.569, *p* = 0.0433) and oxygen use (OR = 1.604, *p* = 0.0027) was positively associated with anti-fibrotic use. Time (log of days) since diagnosis (OR = 1.075, *p* = 0.0477), history of coronary artery disease (OR = 1.796, *p* = 0.0030), presence of pulmonary hypertension (OR = 2.139, *p* = 0.0376), patient clinical trial participation in the prior 12 months (OR = 2.485, *p* = 0.0002), diffusion capacity of the lungs for carbon monoxide (per 10% increase in percent-predicted; OR = 1.138, *p* = 0.0184), anticoagulant use (OR = 2.507, *p* = 0.0028), and enrollment at a registry site in the Midwest region (OR = 1.600, *p* = 0.0446) were associated with pirfenidone use. Anti-fibrotic use varied by registry site. Rates of discontinuation were modest and nearly identical for the two medications with side effects being the most common reason given for discontinuation. Twenty-three percent (23%, 274) of persons with IPF were using or had recently used an immunomodulatory agent.

**Conclusions:**

This analysis provides a detailed characterization of IPF treatment patterns in the US; many users of anti-fibrotic medications may not have qualified for inclusion in clinical trials. More research is needed to understand variations in medical decision-making for use and selection of anti-fibrotic medication.

## Background

Idiopathic pulmonary fibrosis (IPF) is a progressive and often fatal lung disease.^1^ Two anti-fibrotic medications, nintedanib and pirfenidone, slow disease progression. Little is known about prescription patterns for anti-fibrotic medications in a real-world United States (US) setting.

Many persons with IPF were excluded from the clinical trials leading to approval of these medications due to severity of lung function impairment [1, 2]. Specifically, the INPULSIS study of nintedanib excluded persons with less severe lung disease based on diffusion limitation capacity for carbon monoxide (DLCO) > 79%, and the ASCEND study of pirfenidone excluded patients with a forced vital capacity (FVC) > 90% or DLCO> 90%. INPULSIS and ASCEND also excluded patients with more severe disease as indicated by FVC < 50% or DLCO< 30%. For inclusion in the ASCEND trial, patients were also required to walk > 150 m, and patients with chronic obstructive pulmonary disease (COPD) or unstable cardiac disease were excluded. Notable exclusion criteria for the INPULSIS trial included recent myocardial infarction, need for high-dose antiplatelet therapy or need for full dose anticoagulant therapy. Use of an anti-fibrotic in persons with IPF failing to meet clinical trial inclusion criteria is not well described in the US. IPF patient registries are being developed and utilized worldwide for pragmatic research. Published IPF registry studies have provided insights into natural history, patient demographics, diagnostic evaluation practices, quality of life, comorbidities and health care utilization [3–7]. Real world experiences with anti-fibrotic use have also been reported for a single Italian center [8] and in England using administrative data [9]. Additionally, surveys have been used to explore patient and provider views about IPF treatment in Canada, France, Germany, Italy, Spain and the United Kingdom [10]. These surveys suggest that provider characteristics may affect prescription patterns and therefore may be important to patient outcomes. Due to substantial structural differences between the US and global healthcare systems, especially with regard to financing, examination of early use of anti-fibrotics in the US is merited.

This study is a descriptive analysis of early use of anti-fibrotic medications in the United States. We aimed to describe the relationship between patient- and registry site-level characteristics and (a) the likelihood of using anti-fibrotic therapy and (b) the specific anti-fibrotic medication used by patients enrolled in the Pulmonary Fibrosis Foundation Patient Registry (PFF-PR), a multicenter US registry of patients with interstitial lung disease (ILD). This work is intended to generate hypotheses to guide future research regarding variations in use of these medications.

## Methods

### Data source

The PFF-PR cohort includes 1999 well-characterized prevalent and incident ILD patients enrolled at 42 ILD clinics in the US from March 29, 2016 through June 30, 2018. Patients were enrolled and followed by the ILD clinics, which are tertiary care centers in all geographic areas of the US (Additional file 1: Appendix 1). Nine patients who had lung transplants prior to enrollment were excluded from the analysis as well as 7 patients missing diagnosis information. Of the remaining 1983 patients, 1224 patients (61.7%) had IPF. Of this group, there were 1218 patients for which baseline medication information was recorded. IPF diagnosis was confirmed at the registry site with evaluation including at minimum a medical history, physical exam, pulmonary function test (PFT), and computerized tomography scan of the chest (CT). Multi-disciplinary discussion was not required for confirmation of diagnosis. Decisions regarding use of an anti-fibrotic medication were made by the ILD clinic independent of registry participation.

### Study design

Characteristics of persons with IPF and PFF-PR registry sites were analyzed to determine associations with use of (a) any anti-fibrotic medication and (b) nintedanib versus (vs.) pirfenidone. Pirfenidone was arbitrarily chosen as the reference by the independent statistician. Anti-fibrotic medication use was defined as any current or prior use within twelve months of registry enrollment. Use of an anti-fibrotic prior to this period was not recorded.

Patient characteristics were examined as recorded at time of registry enrollment; follow-up data was not examined as part of this analysis. Patient demographic variables of interest were patient age, sex, race, and insurance type. We also examined patient characteristics of specific comorbid diagnoses including COPD, pulmonary hypertension, gastroesophageal reflux disease (GERD), obstructive sleep apnea (OSA), coronary artery disease (CAD), and smoking status. In addition, the following clinical characteristics were included in the analysis: days since IPF diagnosis, presentation at multidisciplinary conference, supplemental oxygen use, concurrent or recent use of an anticoagulant^2^ or non-corticosteroid immunomodulatory medication,^3^ participation in pulmonary rehabilitation, 6-min walk distance (6-MW), FVC percent-predicted [11], DLCO [12], Fatigue severity score [13], Leicester cough score [14], Short-Form Six-Dimension (SF-6D) score, University of California, San Diego (UCSD) shortness of breath score [15], and recent clinical trial participation (current or within the twelve months preceding registry enrollment). Rates and reasons for discontinuation of anti-fibrotic medications were reported.

Characteristics of registry sites were also analyzed. Registry site-level characteristics included for analysis include average and maximum monthly ultraviolet (UV) index,^4^ participation in the CAPACITY, ASCEND or INPULSIS clinical trials for anti-fibrotic medications [16], and geographic region.^5^

### Statistical analysis

Thirty-seven patients were excluded from the second analysis, nintedanib vs. pirfenidone use, because of use of both medications in the prior twelve months. Generalized linear mixed models with a logit link and a binary distribution were used for univariable and multivariable regressions for the outcomes of (a) any anti-fibrotic use among persons with IPF and (b) pirfenidone use (compared to nintedanib use) among anti-fibrotic users. Proportions are reported for categorical variables and means for continuous variables. Odds ratios (OR), coefficients, 95% confidence intervals, and *p*-values are reported for model outputs. The logarithm of the variable days since IPF diagnosis was used to account for the distribution of this variable.

Patient-level variables were chosen for inclusion in the multivariable analysis based on results of the univariable analysis and clinical experience. A combination of backward and forward stepwise selection was used to determine the best fitting multivariable model, using the magnitude of the coefficient, the area under the receiver operating curve, and statistical significance as criteria. The analysis did not find evidence that age, sex, and COPD were confounders or modifiers of the associations with anti-fibrotic usage.

Missing data patterns were examined along with group means across missing data patterns. Multiple imputation was used for sensitivity analysis. For the anti-fibrotic model, twenty imputed datasets were generated using all the variables in the analytic model as well as the following variables: sex, FVC, pulmonary rehabilitation, OSA, COPD and 6-MW. For the nintedanib vs. pirfenidone model, twenty imputed datasets were generated using all the variables in the analytic model as well as the following variables: sex, FVC, pulmonary rehabilitation, UCSD shortness of breath score, and COPD.

A random effects model was used to account for differences in patient and registry site characteristics when evaluating for different likelihoods of (a) anti-fibrotic and (b) pirfenidone prescriptions between registry sites [17]. The random effects model provided a Median Odds Ratio (MOR) which quantifies the variation in anti-fibrotic prescription between sites as an OR. An MOR different than one provides evidence that the likelihood of a patient being prescribed an anti-fibrotic medication varied based on the registry site at which that patient was enrolled. The model also provides the 80% interval odds ratio (IOR-80), a fixed-effects measure for quantification of the effect of cluster-level (registry site) variables. A narrow IOR-80 indicates a small amount of residual variation between sites. For an IOR-80 containing the value one, the area characteristic is not strong compared to the unexplained between-site variation.

Analyses were performed using SAS version 9.4, (SAS Institute, Cary, NC, USA).

## Results

### Cohort demographics and description of missing data

At the time of the analysis, 1224 of 1983 PFF-PR (61.7%) patients had a diagnosis of IPF, of whom 1218 had medication information. There were 740 (60.7%) persons with IPF taking at least one anti-fibrotic medication. Thirty-seven (37) patients reported using both medications in the prior twelve months; 5 patients are listed as having taken both medications concurrently whereas the remaining 32 discontinued one medication and then initiated the other. There were 703 (57.7%) persons with IPF taking a single anti-fibrotic medication. Among those 703 patients, 312 (44.4%) were taking nintedanib and 391 (55.6%) were taking pirfenidone.

71 (9.6%) of anti-fibrotic users had FVC > 90%, and 107 (14.5%) of anti-fibrotic users had severe restrictive disease as indicated by FVC < 50%. Twenty-five patients with diagnoses for COPD, an exclusion criterion for ASCEND, were using pirfenidone. Only four [4] nintedanib patients had DLCO > 80% while five [5] pirfenidone patients had DLCO > 90%. Twenty-five percent (25%, 181) of patients using an anti-fibrotic had DLCO < 30%.

Patients were approximately 76% male with a mean age of seventy years (Table 1). Recent clinical trial participation was relatively uncommon overall (13.5%). Twenty-three percent (23%, 274) of persons with IPF were using or had recently used an immunomodulatory agent. Forty-three percent (43%, 519) of persons with IPF were discussed at multidisciplinary conference.

**Table 1:** Patient characteristics by anti-fibrotic use and by specific medication

Characteristic: count, mean (SD) or Frequency (%)	IPF patients, *n* = 1218		Using an anti-fibrotic, *n* = 703	
	No anti-fibrotic use (*n* = 478)	Anti-fibrotic use (*n* = 740)	Nintedanib (*n* = 312)	Pirfenidone (*n* = 391)
Demographics				
Age	70.8 (8.6)	70.2 (7.6)	69.9 (7.3)	70.6 (7.7)
Male	358 (74.9)	563 (76.1)	238 (76.3)	302 (77.2)
Race: White	432 (90.4)	695 (93.9)	288 (92.3)	373 (95.4)
Black or African American	7 (1.5)	11 (1.5)	6 (1.9)	5 (1.1)
Asian and other	39 (8.2)	34 (4.6)	18 (5.8)	13 (3.3)
Insurance: Private	248 (51.9)	385 (52.0)	167 (53.5)	197 (50.4)
Medicare	181 (37.8)	284 (38.4)	116 (37.2)	153 (39.1)
Medicaid	7 (1.5)	10 (1.4)	4 (1.3)	5 (1.3)
Medicare-Medicaid	1 (0.2)	1 (0.1)	0 (0)	1 (0.2)
Military	6 (1.3)	6 (0.8)	4 (1.3)	2 (0.5)
Other/none/unknown	35 (7.3)	54 (7.3)	21 (6.7)	33 (7.5)
Medical History				
COPD	39 (8.3)	59 (8.0)	31 (10.1)	25 (6.4)
Pulmonary hypertension	23 (4.9)	48 (6.5)	14 (4.6)	31 (8.0)
GERD	79 (16.5)	129 (17.5)	61 (19.6)	59 (15.1)
Obstructive sleep apnea	109 (22.8)	211 (28.5)	85 (27.0)	117 (29.9)
Coronary artery disease	107 (22.8)	194 (26.4)	68 (22.1)	118 (30.3)
Smoking – current or previous	301 (63.0)	475 (64.3)	195 (62.5)	254 (65.1)
Clinical Characteristics				
Days since diagnosis	772.0 (1009.3)	1001.2 (927.3)	879.0 (886.2)	1088.4 (929.4)
Multidisciplinary conference	207 (43.4)	312 (42.2)	135 (43.3)	163 (41.8)
Oxygen use	147 (30.8)	371 (50.1)	148 (47.4)	198 (50.4)
Pulmonary rehabilitation	67 (14.0)	219 (29.6)	93 (29.8)	108 (27.6)
6-min walk distance (m, mean SD)	377.8 (156.7)	357.2 (136.7)	363.6 (138.1)	354.2 (136.9)
FVC (percent-predicted, mean SD)	71 (18)	68 (17)	67 (17)	69 (17)
FVC categorical: > 90%	59 (12.3)	71 (9.6)	31 (9.9)	40 (10.2)
50 to < 90%	328 (68.6)	527 (71.2)	220 (70.5)	278 (71.1)
< 50%	47 (9.8)	107 (14.5)	43 (13.8)	58 (14.8)
DLCO (percent-predicted, mean SD)	45 (17)	40 (17)	38 (16)	41 (18)
DLCO categorical: > 90%	8 (1.7)	8 (1.1)	3 (1.0)	5 (1.3)
80 to < 90%	7 (1.5)	4 (0.5)	1 (0.3)	3 (0.8)
30 to < 80%	310 (64.9)	470 (63.5)	186 (59.6)	265 (67.8)
< 30%	67 (14.0)	181 (24.5)	81 (26.0)	84 (21.5)
Fatigue severity scale (mean, SD)	4.1 (1.8)	4.3 (1.7)	4.2 (1.7)	4.2 (1.7)
Leicester cough score (mean, SD)	16.8 (3.5)	16.4 (3.6)	16.6 (3.7)	16.4 (3.5)
SF-6D score (mean, SD)	0.7 (0.1)	0.7 (0.1)	0.7 (0.1)	0.7 (0.1)
UCSD shortness of breath score (mean, SD)	37.7 (26.8)	42.4 (25.2)	41.8 (24.4)	42.5 (25.8)
Anticoagulant use	43 (9.0)	79 (10.7)	21 (6.7)	51 (13.0)
Immunomodulatory medication use	124 (26.0)	150 (20.3)	70 (22.4)	71 (18.2)
Clinical trial participation in last 12mo.	42 (8.8)	123 (16.6)	30 (9.7)	90 (23.0)

Abbreviations: Idiopathic Pulmonary Fibrosis (IPF); standard deviation (SD); Chronic obstructive pulmonary disease (COPD); gastroesophageal reflux disease (GERD); meters (m); forced vital capacity (FVC); diffusion limitation for carbon monoxide (DLCO); Short-Form Six-Dimension (SF-6D); University of California, San Diego (UCSD); months (mo)

More than 80% of persons with IPF were enrolled at registry sites that had previously participated in clinical trials (Table 2). Anti-fibrotic use was most common in the South US region and least common in the West US sites.

**Table 2:** Registry site characteristics by anti-fibrotic use and by specific medication

	IPF patients, n = 1218		Using an anti-fibrotic, n = 703	
Characteristic: count, mean (SD) or Frequency (%)	No anti-fibrotic use (*n* = 487)	Anti-fibrotic use (n = 740)	Nintedanib (n = 312)	Pirfenidone (n = 391)
Site-level Characteristics				
Region: South	173 (36.2)	332 (44.9)	153 (49.0)	161 (41.2)
Northeast	96 (20.1)	172 (23.2)	73 (23.4)	91 (23.3)
Midwest	97 (20.3)	154 (20.8)	49 (15.7)	99 (25.3)
West	112 (23.4)	82 (11.8)	37 (11.9)	40 (10.2)
Average UV index	5.9 (1.1)	5.9 (1.1)	6.0 (1.1)	5.9 (1.1)
Maximum UV index	9.4 (1.0)	9.4 (1.0)	9.5 (1.1)	9.4 (1.0)
Clinical trial experience – none	59 (12.7)	149 (20.1)	71 (23.1)	72 (18.9)
Pirfenidone only	118 (25.5)	185 (25.5)	69 (22.5)	105 (27.5)
Nintedanib only	20 (4.3)	22 (3.0)	12 (3.9)	8 (2.1)
Both pirfenidone and nintedanib	265 (57.4)	369 (50.1)	155 (50.5)	197 (51.6)
Participation in any trial (versus none)	403 (87.2)	576 (79.5)	236 (76.9)	310 (81.2)

Abbreviations: Idiopathic Pulmonary Fibrosis (IPF); standard deviation (SD); ultraviolet (UV)

DLCO was among the most missing variables with measurements missing for 172 patients (14%) of persons with IPF. Patients who were not taking an anti-fibrotic medication were more likely to be missing FVC and DLCO measurements (9 and 19%, respectively) than patients taking an anti-fibrotic medication (5 and 5%, respectively).

In the best multivariable model (Table 3) for anti-fibrotic use, anti-fibrotic use decreased non-linearly with increased age (OR = 0.974, *p* = 0.0086; age-squared OR = 0.999, *p* = 0.0114). Patients using supplemental oxygen had increased odds (OR 1.604, *p* = 0.0027) of using an anti-fibrotic as did those with recent clinical trial participation (OR = 1.569, *p* = 0.0433). As the log of days from diagnosis increased, the odds of anti-fibrotic use increased, (OR 1.138, *p* < 0.0001). With each 10% increase in percent-predicted of DLCO, the odds of anti-fibrotic use decreased (OR 0.896, *p* = 0.0007). The area under the curve (AUC) for this model was 0.762 (95% CI: 0.732–0.792). These findings were robust compared to the multiple imputation sensitivity analysis. Univariable results are presented in Additional file 1: Appendices 2a and 2b.

**Table 3:** Final multivariable model for anti-fibrotic use

Characteristic:	Coefficient	Odds ratio	95% Confidence Interval	p-value
Age (per year increase)^a^	−0.024	0.974	0.956–0.992	0.0086
Age^2^ (per year increase, squared)^a^	− 0.001	0.999	0.998–0.999	0.0114
Days since diagnosis (per unit increase in log days)	0.129	1.138	1.088–1.191	< 0.0001
Oxygen use	0.472	1.604	1.178–2.183	0.0027
DLCO (percent-predicted, per 10%)	−0.154	0.896	0.785–0.937	0.0007
(Patient) Clinical trial participation in last 12mo.	0.451	1.569	1.014–2.429	0.0433

^a^Age was centered on age 70

Abbreviations: Idiopathic Pulmonary Fibrosis (IPF); diffusion limitation for carbon monoxide (DLCO); months (mo)

In the best multivariable model (Table 4) for anti-fibrotic selection, increased DLCO (per 10% increase in percent-predicted) was associated with greater odds of using pirfenidone (OR 1.138, *p* = 0.0184). The presence of CAD or pulmonary hypertension (cardiovascular disease) were also associated with increased odds of using pirfenidone (ORs 1.796, *p* = 0.0030; OR = 2.139, *p* = 0.0376). Increased days since diagnosis (log) was associated with increased odds of using pirfenidone (OR = 1.074, *p* = 0.0477). Patients seen at Midwest region registry sites were more likely to use pirfenidone in preference to nintedanib than sites in the South region (OR 1.600, *p* = 0.0446). Recent patient clinical trial participation was associated with pirfenidone use (OR = 2.485, *p* = 0.0002). Use of an anticoagulant was associated with pirfenidone use (OR = 2.507, *p* = 0.0028). The AUC for this model was 0.687 (95% CI: 0.646–0.729). These findings were robust compared to the multiple imputation sensitivity analysis (Additional file 1: Appendix 3). Results of the univariable analysis are shown in Additional file 1: Appendices 3a and 3b.

**Table 4:** Final Multivariable Model for pirfenidone (versus nintedanib) use

Characteristic:	Coefficient	Odds ratio	95% Confidence Interval	*P*-value
Days since diagnosis (per unit increase in log days)	0.071	1.074	0.998–1.155	0.0576
Anticoagulant use	0.919	2.507	1.374–4.575	0.0028
Coronary artery disease	0.588	1.800	1.216–2.664	0.0034
Pulmonary hypertension	0.724	2.062	1.000–4.249	0.0499
DLCO (percent-predicted, per 10%)	0.142	1.153	1.033–1.287	0.0115
(Patient) Clinical trial participation in last 12mo.	0.927	2.526	1.553–4.111	0.0002
Midwest region (compared to South)	0.502	1.652	0.983–2.775	0.0579
Northeast region (compared to South)	0.061	1.062	0.658–1.715	0.8038
West region (compared to South)	−0.114	0.892	0.481–1.655	0.7175

Abbreviations: Idiopathic Pulmonary Fibrosis (IPF); diffusion limitation for carbon monoxide (DLCO); months (mo)

### Results of the random effects model

**Fig. 1:**
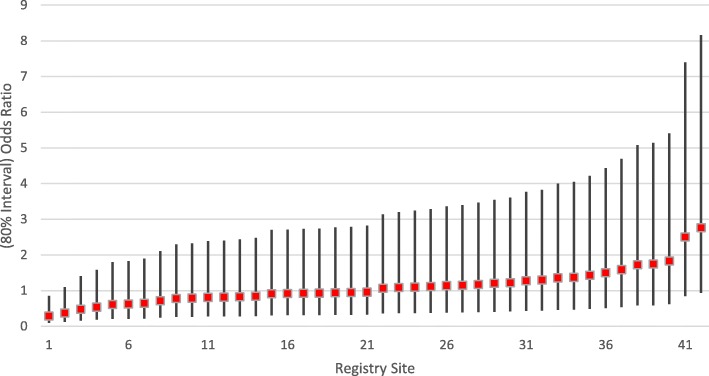
Random Effects Model for Anti-fibrotic Prescription with Patient and Registry Site Variables by Registry Site. In the random effects model for any anti-fibrotic prescription using registry site as a random intercept, there was evidence for variation in propensity to prescribe anti-fibrotics between registry sites after accounting for differences in patient and site characteristics. The IOR-80 (80% interval odds ratio) crossed 1 for all but a single site suggesting that the differences between sites were not explained well by the examined patient and registry site characteristics. The registry site number does not correspond with the order of listing of registry sites in the Additional file 1

For the random effects model of any anti-fibrotic prescription using registry site as a random intercept, the MOR was 2.07 in the null model and 1.77 in the final multivariable model. This provides evidence for variation in propensity to prescribe anti-fibrotics between registry sites after accounting for differences in patient and site characteristics. The IOR-80 crossed 1 for all but a single site (Fig. 1), suggesting that the differences between sites were not explained well by the examined patient and registry site characteristics. Use of a random effects model for the prescription of pirfenidone vs. nintedanib was limited by inadequate statistical power.

### Anti-fibrotic medication discontinuation

Overall, 33 (10.6%) and 42 (10.7%) users of nintedanib and pirfenidone, respectively, discontinued the medications. Reasons given for medication discontinuation are reported in Table 5 with side effects being the most common reason given.

**Table 5:** Anti-fibrotic discontinuation, by medication and reason

Discontinuation Reason	Pirfenidone (n = 391)	Nintedanib (n = 312)
Cost	0	1
Side effects	34	25
Patient preference	2	1
Physician preference	6	6
Total	42	33

## Discussion

This analysis is among the first to examine use of anti-fibrotic medications in the US outside of the clinical trial environment and provides new insights into treatment patterns. Overall, 61% (60.7%) of persons with IPF were using an anti-fibrotic; 58 % (57.7%) were using a single anti-fibrotic. Conversely, 39.3% of persons with IPF were not prescribed one of these medications. Among those using a single medication (703 patients), 312 (44.4%) were taking nintedanib, and 391 (55.6%) were taking pirfenidone. Anti-fibrotic use varied by registry site. Rates of discontinuation were approximately 11% for both medication; the most common reason given was side effects. Many persons with severe IPF who may not have qualified for INPULSIS or ASCEND clinical trials were being treated with an anti-fibrotic in clinical practice. Twenty-three percent (23%, 274) of persons with IPF were using or had recently used an immunomodulatory agent.

The PFF-PR population is similar to the populations of the ASCEND and INPULSIS trials with regards to proportion male, and means of age and severity of lung disease as indicated by FVC (percent-predicted) and DLCO (percent-predicted). The PFF-PR cohort includes patients using an anti-fibrotic medication with more severe and less severe lung disease than those enrolled in the clinical trials at time of enrollment. As the registry does not describe severity of lung function impairment at time of medication initiation, a direct comparison is not possible with the clinical trial populations.

Patient characteristics were also similar in the PFF-PR registry as compared to Australian, German and European registries with regards to FVC, age, and gender [3]. Although anti-fibrotic treatment rates may vary between international registries due to differences in enrollment periods and anti-fibrotic availability, limited comparisons can be made. For example, the German INSIGHTS-IPF registry that began enrolling in 2012 reported pirfenidone use by 44.2% of persons compared to 25.6% use of nintedanib and 32.1% use of pirfenidone in this study.

In general, more severe lung disease – as indicated by lower DLCO and supplemental oxygen use – was associated with anti-fibrotic use. This may be due to providers and patients deferring anti-fibrotic initiation in patients with less severe disease. Such a strategy is not supported by evidence that shows anti-fibrotic use prevents irreversible lung function loss at all levels of disease severity [18]. There was decreasing use of anti-fibrotics with increased age; this may suggest that personalized decision-making results in deferral of anti-fibrotic use in patients of more advanced age for whom loss of future lung function might be of relatively lower concern when balanced against potential side effects and cost. Increased use of anti-fibrotics in recent clinical trial participants may be due to the relative lack of comorbid conditions that allowed these patients to be eligible for clinical trials. Further, clinical trial participants may be different than non-clinical trial participants [19].

One surprising finding is that nearly 40% of persons with IPF were not prescribed one of these disease-altering medications. It is possible that provider unfamiliarity with these newer medications, concerns about side effects, or concerns about financial cost may be reasons for deferral of medication initiation.

Differences in insurance type and status were not associated with anti-fibrotic use or selection. Similarly, cost was rarely listed as a reason for medication discontinuation. It is unlikely that differences among payers across regions was responsible for the significant regional differences in anti-fibrotic use and selection observed.

It is possible that the increased time since diagnosis in anti-fibrotic users is attributable to a survival benefit suggested for anti-fibrotic use [20]. The observation of increased likelihood of pirfenidone use in persons with IPF with longer time since diagnosis also could be related to participation in the CAPACITY trials which were enrolling patients prior to nintedanib trials [2, 16]. Registry site participation in a specific nintedanib or pirfenidone clinical trial was not associated with differences in prescription of that medication.

Registry site was incorporated into a random effects model as a random intercept. Registry site may serve as a proxy for the physician(s) who are an integral component of the shared decision-making process involved in treatment decisions. The random effects model provides evidence of variation in anti-fibrotic use between sites after controlling for patient and registry site characteristics; this variation merits additional study.

There was an association between pirfenidone use in patients with cardiovascular disease (CAD and pulmonary hypertension). This may relate to the initial report of increased myocardial infarction among nintedanib users compared to placebo in the INPULSIS trials [2], the risk of which has been clarified in subsequent analysis [21]. Similarly, anticoagulant use was associated with pirfenidone use. This finding is likely due to differences in exclusion criteria between the INPULSIS and ASCEND trials.

In the final multivariable analysis for nintedanib vs. pirfenidone use, enrollment at a Midwest registry site was associated with higher likelihood of pirfenidone use. To explore this apparent variation by region, the analysis explored for association between the meteorological variable average monthly UV index at the registry site and anti-fibrotic selection, as photosensitivity is a documented adverse effect of pirfenidone [22]. It is possible that patients living in geographic areas with higher average or maximum UV index would be more prone to photosensitivity reactions [23] and therefore avoid use of pirfenidone. However, this association was not seen in the current analysis which could not account specifically for location of patient residence and therefore used registry site as a surrogate. It is possible that unexplained variations in anti-fibrotic selection between registry site exist and that a few proportionately higher-enrolling regional centers affect the overall prescription pattern for the region. Use of a random effects model, limited by inadequate statistical power, could help answer this question. Understanding apparent regional variations in anti-fibrotic selection merits further study.

Twenty-three percent (23%, 274) of persons with IPF were using or had recently used an immunomodulatory agent. Use of these medications for IPF would be discordant with clinical guidelines [24]. One possible reason for prescription of these medications could be treatment of extra-pulmonary disease unrelated to IPF. Another possible use for these medications could be for the proposed disease entity interstitial pneumonia with autoimmune features (IPAF); such a treatment strategy has not been verified by randomized clinical trials. It is also possible that patients were given non-IPF diagnoses and treated with immunomodulatory medications prior to evaluation at the registry site, where the diagnosis was changed to IPF and different treatment recommendations given.

Strengths of this study include analysis of detailed patient information for a cohort of well-characterized persons with IPF. Missing data was minimal and where present, such as for missing values of DLCO, is likely explained by the known difficulty in completing DLCO measurement for patients with the worst lung function [25]. This study benefits from observation of patients in a more real-world setting to evaluate relationships between patient- and registry site-level characteristics and anti-fibrotic use and selection. Use of the random effects model explores the influence of registry site, as a proxy for provider, on anti-fibrotic use. Additionally, the analysis of average UV index and effect on anti-fibrotic selection and the relationship between prior clinical trial participation by a center on subsequent prescription are unique. Inclusion of patients currently or recently participating in clinical trials does not invalidate the analysis as the period of PFF-PR enrollment and entry data gathering did not overlap with the time periods of the ASCEND, INPULSIS or CAPACITY trials.

Limitations include lack of provider-specific information, which required use of registry site as a proxy for physician in this study. However, a single influential provider may influence peer and trainee practice indirectly and directly through participation in multidisciplinary conferences, a core component in the care of persons with IPF. Limitation of analysis to registry sites, of which many were university settings, might limit generalizability. Ultimately, the accuracy of the clinical IPF diagnosis at the enrolling registry site cannot be verified. Confidence in the diagnosis would be increased by more frequent use of multidisciplinary discussion, reported in the registry for 43% of persons with IPF. This analysis only evaluated relationships between characteristics and medication use at time of enrollment to the registry; information regarding anti-fibrotic use prior to 12 months preceding registry enrollment was not collected and subsequent anti-fibrotic use was not analyzed. Statistical power limited the performance of the random effects model to the analysis of nintedanib vs. pirfenidone use which might have helped explain observed regional variations in anti-fibrotic utilization.

## Conclusions

At time of PFF-PR enrollment, 61 % (60.7%) of persons with IPF were using either one or both anti-fibrotic medications; 39% of patients potentially helped by these medications were not prescribed one of the medications. Some registry patients using an anti-fibrotic may have been excluded from clinical trial participation due to having too severe disease, as measured by DLCO. In this study, more severe lung disease, as indicated by worse DLCO and oxygen use, was associated with anti-fibrotic use. There was variation in odds of anti-fibrotic medication use between registry sites that was incompletely explained by examined characteristics. Pirfenidone was used by a small majority (55.6%) of persons with IPF and was associated with patient history of cardiovascular disease, anticoagulant use, recent clinical trial participation, and enrollment at a Midwest region registry site. Twenty-three percent (23%, 274) of persons with IPF were using or had recently used an immunomodulatory agent. Compared to studies based on a limited number of clinical sites or a single payer system, this analysis provides a more detailed and inclusive characterization of US treatment patterns. Use in the US may be different than in international settings due to structural differences in health care financing. More research is needed to better understand variations in medical decision-making regarding use, including at different stages of disease severity, and selection of anti-fibrotic medication.

## Supplementary information

**Additional file 1: Appendix 1.** List of PFF-PR Sites. **Appendix 2a**. Univariable associations between patient characteristics and anti-fibrotic use. **Appendix 2b.** Univariable associations between registry site characteristics and anti-fibrotic use. **Appendix 3a.** Univariable associations between patient characteristics and pirfenidone (versus nintedanib) use. **Table 3b.** Univariable associations between registry site characteristics and pirfenidone (versus nintedanib) use.

## Data Availability

All data generated or analysed during this study are included in this published article [and its supplementary information files].

## Abbreviations

6-MW: 6-min walk distance
AUC: Area under the curve
CAD: Coronary artery disease
CI: Confidence interval
COPD: Chronic obstructive pulmonary disease
CT: Computerized tomography scan of the chest
DLCO: Diffusion limitation for carbon monoxide
FVC: Forced vital capacity
GERD: Gastroesophageal reflux disease
ILD: Interstitial lung disease
IOR-80: 80% interval odds ratio
IPAF: Interstitial pneumonia with autoimmune features
IPF: Idiopathic pulmonary fibrosis
m: Meters
mo.: Months
MOR: Median odds ratio
OR: Odds ratios
OSA: Obstructive sleep apnea
PFF-PR: Pulmonary Fibrosis Foundation Patient Registry
PFT: Pulmonary function test
SD: Standard deviation
SF-6D: Short-Form Six-Dimension
UCSD: University of California, San Diego
US: United States
UV: Ultraviolet

## Footnotes

1 *Prior Publication:* Limb, S., et al. Description and Predictors of Antifibrotic Medication Use Among Patients Enrolled in the Pulmonary Fibrosis Foundation Patient Registry (PFF-PR). American Journal of Respiratory and Critical Care Medicine 2018;197:A1627.

2 Includes current or recent (within the prior 12 months) use of coumarins, heparin, Factor Xa inhibitors, or direct thrombin inhibitors

3 Includes current or recent (within the prior 12 months) use of abatacept, adalimumab, azathioprine, belimumab, cyclophosphamide, cyclosporin A, D-penicillamine, etanercept, gold, golimumab, hydroxychloroquine, infliximab, leflunomide, methotrexate, mycophenolate, rituximab, sulfasalazine, tacrolimus, tocilizumab,

4 Determined as the geometric mean of average monthly UV index for the city as reported by “www.weather-us.com” on 2/27/2019. For the few geographic locations without average UV index reported, data was substituted with that of a nearby city

5 Regions were assigned as follows: **West** – Arizona, California, Colorado, Utah, Washington; **Midwest** – Illinois, Kansas, Michigan, Minnesota, Ohio; **Northeast** - Connecticut, Massachusetts, New York, Pennsylvania; and **South** - Alabama, Florida, Georgia, Kentucky, Louisiana, Maryland, North Carolina, South Carolina, Tennessee, Texas, and Virginia.
