# Supplementary material for: Patient and site characteristics associated with pirfenidone and nintedanib use in the United States; an analysis of idiopathic pulmonary fibrosis patients enrolled in the Pulmonary Fibrosis Foundation Patient Registry
Source: Respir Res. 2020 Feb 10;21:48. doi: 10.1186/s12931-020-1315-4 (PMC7011239; doi:10.1186/s12931-020-1315-4)
Supplement: Supplementary file 1 — Additional file 1: Appendix 1. List of PFF-PR Sites. Appendix 2a. Univariable associations between patient characteristics and anti-fibrotic use. Appendix 2b. Univariable associations between registry site characteristics and anti-fibrotic use. Appendix 3a. Univariable associations between patient characteristics and pirfenidone (versus nintedanib) use. Table 3b. Univariable associations between registry site characteristics and pirfenidone (versus nintedanib) use. [file 12931_2020_1315_MOESM1_ESM.docx]

Additional file

**Appendix 1: List of PFF-PR Sites**

| 1 | University of Alabama at Birmingham | Birmingham | AL |
| --- | --- | --- | --- |
| 2 | Dignity Health St. Joseph's Hospital and Medical Center | Phoenix | AZ |
| 3 | The University of Arizona | Tucson | AZ |
| 4 | UCLA, David Geffen School of Medicine | Los Angeles | CA |
| 5 | University of California at San Francisco | San Francisco | CA |
| 6 | Stanford University | Stanford | CA |
| 7 | National Jewish Health | Denver | CO |
| 8 | Yale School of Medicine | New Haven | CT |
| 9 | University of Miami | Miami | FL |
| 10 | Piedmont Healthcare | Austell | GA |
| 11 | Northwestern University | Chicago | IL |
| 12 | University of Chicago | Chicago | IL |
| 13 | The University of Kansas Medical Center | Kansas City | KS |
| 14 | University of Louisville | Louisville | KY |
| 15 | Tulane University | New Orleans | LA |
| 16 | University of Maryland Medical Center | Baltimore | MD |
| 17 | Johns Hopkins University - School of Medicine | Baltimore | MD |
| 18 | Massachusetts General Hospital | Boston | MA |
| 19 | University of Michigan | Ann Arbor | MI |
| 20 | Regents of the University of Minnesota Twin Cities | Minneapolis | MN |
| 21 | Mayo Clinic | Rochester | MN |
| 22 | Washington University School of Medicine | St. Louis | MO |
| 23 | Columbia University Medical Center | New York | NY |
| 24 | Weill Cornell Medical College | New York | NY |
| 25 | University of Rochester | Rochester | NY |
| 26 | Stony Brook University Hospital | Stony Brook | NY |
| 27 | Duke University Medical Center | Durham | NC |
| 28 | University of Cincinnati | Cincinnati | OH |
| 29 | The Ohio State University | Columbus | OH |
| 30 | Pennsylvania State University | Hershey | PA |
| 31 | University of Pennsylvania | Philadelphia | PA |
| 32 | Temple University Health System | Philadelphia | PA |
| 33 | University of Pittsburgh | Pittsburgh | PA |
| 34 | Medical University of South Carolina | Charleston | SC |
| 35 | Vanderbilt University | Nashville | TN |
| 36 | University of Texas Southwestern Medical Center | Dallas | TX |
| 37 | The University of Texas Health Science Center at Houston | Houston | TX |
| 38 | UTHSC-San Antonio | San Antonio | TX |
| 39 | University of Utah | Salt Lake City | UT |
| 40 | University of Virginia Interstitial Lung Disease Clinic | Charlottesville | VA |
| 41 | Inova Fairfax Hospital | Falls Church | VA |
| 42 | University of Washington | Seattle | WA |

**Appendix 2a: Univariable associations between patient characteristics and anti-fibrotic use**

| **Characteristic:** | **Odds ratio** | **95% Confidence Interval** | **p-value** |
| --- | --- | --- | --- |
| **Demographics** |  |  |  |
| Age | 0.988 | 0.973-1.004 | 0.1362 |
| Male | 1.192 | 0.896-1.585 | 0.2274 |
| Race: White | (reference) |  |  |
| Black or African American | 0.898 | 0.337-2.398 | 0.8306 |
| Asian and other | 0.697 | 0.413-1.178 | 0.1778 |
| Insurance: Private* | (reference) |  |  |
| Medicare | 0.929 | 0.695-1.241 | 0.6160 |
| Medicaid | 1.267 | 0.441-3.639 | 0.6605 |
| Medicare-Medicaid | 0.722 | 0.043-12.133 | 0.8209 |
| Military | 0.640 | 0.190-2.153 | 0.4703 |
| Other/none/unknown | 0.931 | 0.518-1.674 | 0.8109 |
| **Medical History** |  |  |  |
| COPD | 0.891 | 0.567-1.401 | 0.6182 |
| Pulmonary hypertension | 1.246 | 0.722-2.150 | 0.4300 |
| GERD | 0.994 | 0.705-1.402 | 0.9746 |
| Obstructive sleep apnea | 1.299 | 0.977-1.727 | 0.0718 |
| Coronary artery disease | 1.053 | 0.789-1.405 | 0.7244 |
| Smoking – current or previous | 1.017 | 0.785-1.316 | 0.8987 |
| **Clinical Characteristics** |  |  |  |
| Days since diagnosis (log) | 1.159 | 1.113-1.207 | <0.0001** |
| Multidisciplinary conference | 1.048 | 0.749-1.467 | 0.7823 |
| Oxygen use | 2.194 | 1.689-2.850 | <0.0001** |
| Pulmonary rehabilitation | 2.092 | 1.512-2.894 | <0.0001** |
| 6-minute walk distance (per 100 m) | 0.912 | 0.831-1.000 | 0.0510 |
| FVC (percent-predicted, per 10%) | 0.915 | 0.849-0.985 | 0.0180** |
| FVC categorical: >90% | (reference) |  |  |
| 50% to <90% | 1.214 | 0.815-1.808 | 0.3410 |
| <50% | 1.793 | 1.068-3.013 | 0.0273** |
| DLCO (percent-predicted, per 10%) | 0.809 | 0.745-0.880 | <0.0001** |
| DLCO categorical: >90% | (reference) |  |  |
| 80% to <90% | 0.812 | 0.153-4.317 | 0.8068 |
| 30% to <80% | 1.742 | 0.607-4.998 | 0.3018 |
| <30% | 3.025 | 1.022-8.953 | 0.0456** |
| Fatigue severity scale | 1.039 | 0.968-1.115 | 0.2911 |
| Leicester cough score | 0.976 | 0.942-1.010 | 0.1677 |
| SF-6D score | 0.658 | 0.207-2.092 | 0.4774 |
| UCSD shortness of breath score | 1.006 | 1.001-1.011 | 0.0134** |
| Anticoagulant use | 1.161 | 0.764-1.763 | 0.4841 |
| Immunomodulatory medication use | 0.755 | 0.561-1.014 | 0.0621 |
| Clinical trial participation in last 12mo. | 1.902 | 1.263-2.865 | 0.0021** |

**denotes statistical significance for an alpha < 0.05

Abbreviations: Idiopathic Pulmonary Fibrosis (IPF); Chronic obstructive pulmonary disease (COPD); gastroesophageal reflux disease (GERD); meters (m); forced vital capacity (FVC); diffusion limitation for carbon monoxide (DLCO); Short-Form Six-Dimension (SF-6D); University of California, San Diego (UCSD); months (mo.)

**Appendix 2b: Univariable associations between registry site characteristics and anti-fibrotic use**

| **Characteristic:** | **Odds ratio** | **95% Confidence Interval** | **p-value** |
| --- | --- | --- | --- |
| Region: South | (reference) |  |  |
| Northeast | 1.035 | 0.517 – 2.071 | 0.9236 |
| Midwest | 0.847 | 0.419 - 1.713 | 0.6444 |
| West | 0.426 | 0.205 - 0.885 | 0.0222** |
| Average UV index | 0.948 | 0.741 – 1.213 | 0.6719 |
| Maximum UV index | 0.968 | 0.749 – 1.250 | 0.8008 |
| Clinical trial experience – none | (reference) |  |  |
| Pirfenidone only | 0.564 | 0.254 – 1.254 | 0.1601 |
| Nintedanib only | 0.377 | 0.095 – 1.502 | 0.1666 |
| Both pirfenidone and nintedanib | 0.432 | 0.205 – 0.909 | 0.0271** |
| Participation in any trial (versus none) | 0.474 | 0.240 – 0.938 | 0.0320** |

**denotes statistical significance for an alpha < 0.05

Abbreviations: ultraviolet (UV)

**Appendix 3a: Univariable associations between patient characteristics and pirfenidone (versus nintedanib) use**

| **Characteristic:** | **Odds ratio** | **95% Confidence Interval** | **p-value** |
| --- | --- | --- | --- |
| **Demographics** |  |  |  |
| Age | 1.014 | 0.994-1.035 | 0.1773 |
| Male | 1.067 | 0.747-1.526 | 0.7203 |
| Race: White | (reference) |  |  |
| Black or African American | 0.612 | 0.182-12.058 | 0.3655 |
| Asian and other | 0.550 | 0.262-1.156 | 0.2176 |
| Insurance: Private | (reference) |  |  |
| Medicare | 1.243 | 0.887-1.743 | 0.2066 |
| Medicaid | 1.125 | 0.285-4.449 | 0.8661 |
| Medicare-Medicaid | -- | -- | -- |
| Military | 0.384 | 0.067-2.199 | 0.2818 |
| Other/none/unknown | 1.291 | 0.668-2.495 | 0.4468 |
| **Medical History** |  |  |  |
| COPD | 0.611 | 0.348-1.075 | 0.0875 |
| Pulmonary hypertension | 1.839 | 0.946-3.574 | 0.0724 |
| GERD | 0.764 | 0.509-1.146 | 0.1924 |
| Obstructive sleep apnea | 1.198 | 0.855-1.678 | 0.2942 |
| Coronary artery disease | 1.533 | 1.078-2.179 | 0.0175** |
| Smoking – current/previous | 1.103 | 0.804-1.514 | 0.5430 |
| **Clinical Characteristics** |  |  |  |
| Days since diagnosis (log) | 1.093 | 1.024-1.166 | 0.0007** |
| Multidisciplinary conference | 0.853 | 0.596-1.221 | 0.3850 |
| Oxygen use | 1.127 | 0.829-1.531 | 0.4453 |
| Pulmonary rehabilitation | 0.934 | 0.664-1.314 | 0.6960 |
| 6-minute walk distance (per 100m) | 0.984 | 0.884-1.096 | 0.7734 |
| FVC (percent-predicted, per 10%) | 1.068 | 0.975-1.171 | 0.1568 |
| FVC categorical: >90% | (reference) |  | (ref) |
| 50% to <90% | 0.960 | 0.576-1.601 | 0.8755 |
| <50% | 1.077 | 0.539-1.882 | 0.9818 |
| DLCO (percent-predicted, per 10%) | 1.088 | 0.984-1.204 | 0.1008 |
| DLCO categorical:>90% | (reference) |  |  |
| 80% to <90% | 1.718 | 0.116-25.485 | 0.6935 |
| 30% to <80% | 0.837 | 0.195-3.595 | 0.8106 |
| <30% | 0.611 | 0.139-2.675 | 0.5122 |
| Fatigue severity scale | 0.999 | 0.914-1.092 | 0.9832 |
| Leicester cough score | 0.986 | 0.945-1.029 | 0.5149 |
| SF-6D score | 0.529 | 0.119-2.349 | 0.4018 |
| UCSD shortness of breath score | 1.001 | 0.995-1.008 | 0.6795 |
| Anticoagulant use | 2.275 | 1.319-3.925 | 0.0032** |
| Immunomodulatory medication use | 0.178 | 0.539-1.155 | 0.2232 |
| Clinical trial participation in last 12 mo. | 2.733 | 1.735-4.304 | <0.0001** |

**denotes statistical significance for an alpha < 0.05

Abbreviations: Idiopathic Pulmonary Fibrosis (IPF); Chronic obstructive pulmonary disease (COPD); gastroesophageal reflux disease (GERD); meters (m); forced vital capacity (FVC); diffusion limitation for carbon monoxide (DLCO); Short-Form Six-Dimension (SF-6D); University of California, San Diego (UCSD); months (mo.)

**Table 3b: Univariable associations between registry site characteristics and pirfenidone (versus nintedanib) use**

| **Characteristic:** | **Odds ratio** | **95% Confidence Interval** | **p-value** |
| --- | --- | --- | --- |
| Region: South | (reference) |  |  |
| Northeast | 1.276 | 0.800 – 2.033 | 0.3053 |
| Midwest | 1.969 | 1.205 – 3.218 | 0.0070** |
| West | 1.042 | 0.587 – 1.849 | 0.8886 |
| Average UV index | 0.864 | 0.727 – 1.029 | 0.1021 |
| Maximum UV index | 0.883 | 0.736 – 1.059 | 0.1796 |
| Clinical trial experience – none | (reference) |  |  |
| Pirfenidone only | 0.776 | 0.545 – 1.754 | 0.9392 |
| Nintedanib only | 0.448 | 0.147 – 1.368 | 0.1583 |
| Both pirfenidone and nintedanib | 0.978 | 0.451 – 1.334 | 0.3589 |
| Participation in any trial (versus none) | 1.360 | 0.838 – 2.205 | 0.2125 |

**denotes statistical significance for an alpha < 0.05

Abbreviations: Idiopathic Pulmonary Fibrosis (IPF); ultraviolet (UV)
